# Exploring the autism and functional neurological disorder association: Considerations from biopsychosocial, neuropsychological and computational models

**DOI:** 10.1177/13623613251393504

**Published:** 2025-12-17

**Authors:** Richard H Cole, Lily Smythe, Mark J Edwards, Francesca Happé, Timothy R Nicholson

**Affiliations:** 1 King’s College London, UK

**Keywords:** alexithymia, autism, functional neurological disorder, interoception, predictive processing

## Abstract

Evidence is accumulating regarding an association between autism and functional neurological disorder, a common cause for a wide range of neurological symptoms affecting motor, sensory and cognitive systems. Symptoms can include paralysis, tremors, sensory disturbance, vision loss and dizziness. Functional neurological disorder exists at the complex intersection of physical and mental health, neurology and psychiatry, and body and mind. Despite a recent resurgence in clinical and scientific interest, functional neurological disorder has lagged behind other causes of neurological symptoms in research, service development and acceptance. The nature of the association between autism and functional neurological disorder remains uncertain, but several plausible mechanisms can be identified from overlapping areas of research, highlighting endogenous factors such as atypical interoception, motor function, emotional processing and sensorimotor integration, alongside exogenous influences including adversity, healthcare inequality and stigma. This review first provides an overview of functional neurological disorder through various explanatory frameworks before applying biopsychosocial, neuropsychological and computational perspectives to conceptualise its intersection with autism. It then considers how this association might be understood and explores how services could be adapted to better recognise and support autistic individuals with functional neurological disorder across the diagnostic and treatment pathway.

**Lay Abstract:**

Functional neurological disorder causes real and often disabling symptoms, such as seizures, paralysis, tremors or sensory changes, even though standard medical tests do not show physical damage to the nervous system. Research suggests that autistic people are more likely to experience functional neurological disorder than their non-autistic peers, but the reasons for this are not yet understood. This article explores why autism and functional neurological disorder might occur together. It draws on research into how the brain processes body signals (like pain or movement), handles emotions and responds to uncertainty. It also looks at life experiences that affect health, including trauma, barriers to healthcare and stigma. This article shows that both internal factors (such as differences in movement, emotional awareness and sensory processing) and external factors (such as stress, inequality and misdiagnosis) may increase the chances of functional neurological disorder in some autistic individuals. Several models are introduced to help explain how these influences might interact. Finally, this article outlines how healthcare services could better support autistic people with functional neurological disorder. It encourages functional neurological disorder services to adapt communication styles, provide appropriate adjustments and include autistic voices in research and treatment planning to improve care and outcomes.

## Introduction

Recent cross-sectional and meta-analytic evidence suggests a heightened prevalence of autistic traits or autism spectrum disorder (ASD) (herein referred to as autism) among individuals with functional neurological disorder (FND) (Smythe et al., 2025; Tamilson et al., 2025; Vickers et al., 2024) and, more broadly, those experiencing functional somatic syndromes (FSSs) such as chronic fatigue syndrome (CFS) and chronic pain (Grant et al., 2022).

Functional neurological symptoms do not arise from structural damage to the nervous system (e.g., stroke) but are believed to stem from maladaptive alterations in neural networks involved in attention, interoception and sensorimotor processing (see Supplementary Material for a glossary of terms used in this article) (Hallett et al., 2022). Health events (injury, acute illness, medical procedures) and stressors are common precipitants, with a range of predisposing factors across physical health, mental health and psychosocial domains (Mavroudis et al., 2025).

FND presents with genuinely experienced, often severe, involuntary neurological symptoms, such as weakness, tremors and seizure-like episodes, commonly existing alongside neurological and psychiatric disorders, as well as chronic pain syndromes such as fibromyalgia (Hallett et al., 2022; Popkirov et al., 2019; Steinruecke et al., 2024), all of which occur more frequently in the autistic population (Croen et al., 2015; Grant et al., 2022; Rosen et al., 2018).

Similar to FND, FSS, such as fibromyalgia, is characterised by persistent bodily symptoms without structural pathology (Henningsen et al., 2007). The terminology of FSS usually reflects the body system of the main complaint (e.g., irritable bowel syndrome), yet these conditions all involve nervous system processes such as pain perception, autonomic regulation and visceral function.

While specific changes between the brain and particular organ systems (e.g., gut-brain axis) may contribute especially to certain FSS subtypes (Mayer et al., 2023), growing evidence suggests overlapping clinical and aetiological features between FND and FSS, including attentional dysregulation and interoceptive alterations (Sharp et al., 2021; Teodoro et al., 2018). While this article focuses on FND, there is likely a mechanistic convergence contributing to the increased prevalence of both FND and FSS in autistic people, requiring further investigation.

The heightened risk of FND in autism highlights an under-recognised and under-resourced contributor to disability, stigma and reduced quality of life in autistic people. Given the female predominance and typical adult onset of FND (female-to-male ratio 3:1; Finkelstein et al., 2025), autism may go unrecognised in many affected individuals. Smythe et al. (2025) found that 47% of autistic people within an FND cohort (*n* = 220,312) were diagnosed with autism only after their FND diagnosis. As undiagnosed autism is associated with substantial psychosocial burden (Lai & Baron-Cohen, 2015), it may act both independently and interactively with other stressors to increase vulnerability to FND.

The cause or nature of the autism–FND association remains unclear. A causal connection may exist, driven by underlying mechanisms in autism contributing directly to FND vulnerability. Given the genetic and neurodevelopmental basis of autism (Krishnan et al., 2016), a causal pathway from FND to autism appears unlikely, although clinical contact for FND may hasten a first diagnosis of autism in adulthood. Alternatively, the association may be artefactual, arising from conceptual overlap or covarying factors that increase the likelihood of both conditions occurring together (e.g., atypical interoception and emotion processing; Ricciardi et al., 2016; Williams et al., 2022).

After providing an overview of FND, this perspective piece will explore endogenous and exogenous factors relevant to the autism–FND intersection by drawing on distinct bodies of research. We will then apply a computational conceptualisation to the relationship, where, within a predictive processing model, abnormal high-level expectations or predictions are hypothesised to disrupt perception and voluntary movement, interacting with interoception and emotional experiences.

Finally, it will consider how to conceptualise the association and the clinical implications. Instead of presuming a definitive link or endeavouring to address all pertinent constructs exhaustively, this article provides a theoretical synthesis of potential contributing factors.

### Overview of FND

#### Symptoms

The diversity of functional neurological symptoms highlights the capacity of cognitive, motor and sensory systems to experience malfunction without direct injury, as observed in functional cognitive disorder, functional motor symptoms and functional sensory loss, respectively. Reflecting this, FND commonly spans multiple symptom domains: functional seizures (FSs), for instance, commonly encompass involuntary movements (tremors, jerking), sensory changes (prodromal numbness, vision alterations) and cognitive dysfunction (altered awareness and dissociation) (Hallett et al., 2022; Pick et al., 2019).

Functional symptoms may appear to mimic neurological symptoms arising from structural pathology (so-called ‘organic’ symptoms); however, they can be differentiated by careful clinical examination, highlighting variability in symptom characteristics (or perception thereof) and improvement with distraction (Kozlowska & Mohammad, 2022). For example, a functional hand tremor may cease or change its frequency during a distraction manoeuvre, unlike a tremor due to Parkinson’s disease, and functional leg weakness might improve when attention is fully shifted to the unaffected leg or during automatic movement (e.g., on a treadmill) (Hallett et al., 2022).

Healthcare practitioners with a limited understanding of FND may too readily consider the only explanation for such signs to be feigning; however, they are genuine and distinct from feigned symptoms, as supported by experimental evidence and clinical and epidemiological studies (Edwards et al., 2023). The use of such positive clinical signs should facilitate the diagnosis of FND and help avoid a ‘diagnosis of exclusion’ approach, which can result in unnecessary investigations, cause patient distress and increase the risk of misattributing non-pathological findings as explanations for symptoms.

FND can occur alongside neurological disorders (e.g., FSs with epilepsy), but there may also be a layering effect, where the same symptom may be contributed to by both nervous system pathology and worsened through mechanisms related to FND, such as attention. A functional overlay may be revealed through partial symptom improvement with distraction or suggestion (Kurtis & Pareés, 2021).

#### Understanding and conceptualisation

The terminology used to describe FND through history reflects evolving conceptualisations of the disorder, from ‘hysteria’ to ‘conversion disorder’ and ‘psychosomatic’ or ‘medically unexplained’ symptoms (see Box 1). Currently, FND is classified in the *Diagnostic and Statistical Manual of Mental Disorders* (5th ed., text rev.; DSM-5-TR) as Functional Neurological Symptom Disorder (Conversion Disorder) and in the International Classification of Diseases (ICD)-11 as Dissociative Neurological Symptom Disorder.

**Box 1.** Evolution of FND Terminology.

• **Ancient Views:** Symptoms were attributed to supernatural causes (Egypt, ~1900 BC) or ‘hysteria’ linked to a ‘wandering womb’ (Greece, ~400 BC) • **19th Century:** Clinicians such as Pierre Briquet (1859) and Russell Reynolds (1869) described symptoms arising from interactions between emotion, ideas and bodily experience. Briquet also highlighted the role of stress, trauma and injury as predisposing and precipitating factors (Stone, 2025). Charcot identified hysteria as a neurological condition, later reframed by Freud as conversion disorder, initiating a long-standing period of dualistic conceptualisation • **20th Century:** Terms such as psychosomatic and somatoform emerged, later replaced by medically unexplained symptoms • **Modern Classification:** FND is now listed in DSM-5-TR as functional neurological symptom disorder. ‘Functional’ emphasises nervous system dysfunction without structural pathology, while ‘dissociative’ highlights psychological processes

The biopsychosocial model views FND as resulting from a combination of biological, psychological and social factors, framed as predisposing, precipitating and perpetuating influences. These may include trauma, illness or injury, and how such experiences are shaped by personal meaning, cultural context, coping style and prior experiences (Joos et al., 2023). This model supports a holistic and individualised understanding of symptoms.

The neuropsychological model links symptoms to psychophysical constructs (theoretical entities) such as interoception, embodiment and emotion, and network connectivity. It offers a mechanistic explanation for symptoms while adopting a transdiagnostic view, framing FND as a dimensional variation in these processes rather than a discrete disorder. For example, heightened connectivity between emotional and motor circuits may disrupt voluntary movement (Pick et al., 2019).

The computational approach to FND explains symptoms as arising from disruptions in how the brain predicts and interprets sensory information. Rather than responding passively to sensory input, the brain constructs experience by generating predictions, called priors, about exteroceptive, interoceptive and proprioceptive signals, and updating them based on prediction errors (see Box 2). In FND, perceptual experience may be shaped more by maladaptive priors that misrepresent the body’s state than by accurate ones calibrated to reflect incoming sensory data (Edwards et al., 2012).

**Box 2.** Predictive Perception Terminology.

**Predictive Perception:** The brain constructs hierarchical generative models, shaped by prior experiences, to predict internal (bodily) and external (environmental) states. Incoming sensory inputs (exteroceptive, proprioceptive, interoceptive) are compared to these predictions (priors). Mismatches generate prediction errors (PEs), which update the model and shape perception (Ma, 2019) **Precision Weighting (PW):** PW determines the relative influence of priors versus PEs based on their estimated reliability. It modulates perceptual experience by adjusting neural gain, guided by neuromodulators like dopamine and acetylcholine (Limanowski et al., 2024). Adaptive PW filters out irrelevant or noisy inputs, supporting efficient perception and action. **Active Inference:** AI extends predictive processing by integrating action. When priors predict movement or physiological change, high precision on the prior may lead the system to adjust sensory input (e.g., proprioception) rather than revise the model, minimising PE via action rather than perception. AI also helps reduce sensory uncertainty by actively sampling the environment (Friston, 2014). **Allostasis:** Predictive processing supports allostasis, the anticipatory regulation of bodily states. For example, a prior for increased heart rate in anticipation of running triggers autonomic changes to match the expectation, minimising future prediction error and optimising physiological efficiency (McEwen & Gianaros, 2016).

For example, functional sensory loss or tremor may result from overly precise predictions that override contradictory bodily signals, producing numbness or involuntary movement without structural damage. Attention plays a key role by increasing the precision of symptom-related priors, reinforcing the mismatch between priors and actual input, but also offering a therapeutic strategy of improvement with distraction (Perez et al., 2021).

### Framing an autism and FND intersection

#### The biopsychosocial framework as applied to autism and FND

Many biopsychosocial factors in FND research are well-studied in the autism literature, such as interpersonal adversity, social inequality and barriers to healthcare (Raymaker et al., 2017; Stewart et al., 2022), autonomic and interoceptive dysfunction (Owens et al., 2021; Proff et al., 2022), affective dysregulation (McDonnell et al., 2019) and sensory processing and attention modulation differences (Demetriou et al., 2018; He et al., 2023). This section explores several factors which may be significant in this framework for understanding the autism–FND association.

##### Biological factors

The relatively high prevalence of neurological disorders in the autistic population, such as epilepsy (14.2%), migraine (7.2%) and those affecting motor function (Bell et al., 2019; Millman et al., 2023; Pan et al., 2021), may influence the risk of developing FND through multiple pathways. These include heightened bodily attention, altered illness beliefs, reduced resilience, increased exposure to medical interventions and distressing somatic experiences, and potential disruptions to sensorimotor integration.

Living with chronic, episodic disorders such as epilepsy or migraine, characterised by distressing pre-ictal or premonitory sensory phenomena and unpredictable symptom recurrence, may be particularly destabilising for autistic individuals, where reduced tolerance of uncertainty and heightened sensory reactivity are more prevalent (He et al., 2023; South & Rodgers, 2017). These factors may, in turn, amplify somatic hypervigilance, health-related anxiety and expectancy biases towards pain and dysfunction.

Motor dysfunction affects a significant proportion of the autistic population, across multiple stages of motor control, including planning, execution and real-time adjustment (Shafer et al., 2021). Research suggests the relative risk of motor impairment in the autistic population to be 22 times higher than in the general population (Bhat, 2021) and may, in some, be associated with the degree of core differences in social, communication and cognitive domains, and repetitive behaviours (see Estes et al., 2015; Hannant et al., 2016; LeBarton & Iverson, 2013; Ravizza et al., 2013; Shafer et al., 2021).

While speculative, there may be more direct mechanistic pathways through which motor impairments could contribute to increased risk of FND. For example, Mostofsky et al. (2009) reported that autistic children in their study were more likely to demonstrate diffusely decreased connectivity across the motor execution network relative to controls and relative hyperactivation of the supplementary motor area (SMA) and hypoactivation of the cerebellum during voluntary movement finger-tapping tasks. They proposed this may reflect difficulty shifting motor execution from cortical regions associated with effortful conscious control to regions associated with habitual execution, an alteration relevant to proposed mechanisms in motor FND, where increased attention to automatic processes may paradoxically impede function (Baek et al., 2017).

Some autistic individuals may also be more likely to exhibit impairments in visuomotor integration, as demonstrated by Shafer et al. (2021), who attributed these to difficulties integrating sensory information in order to flexibly guide motor output. Lidstone and Mostofsky (2021) suggested an over-reliance on internal proprioceptive signals at the expense of external visual feedback. Such difficulties in dynamically updating motor priors in response to changing sensory inputs may be conceptualised computationally as maladaptive precision weighting dynamics and error-based corrective feedback. This overlaps with key mechanistic models of motor FND, which implicate abnormalities across multiple stages of action control, including motor generation, automaticity and the sense of agency (Matar et al., 2024).

Sensory processing is an important but difficult area to compare directly between autism and FND, given inconsistent definitions across studies (e.g., sensory thresholds, physiological reactivity or affective appraisal; He et al., 2023), considerable heterogeneity within each group, and intraindividual variability across time and context. Nonetheless, sensory over-responsivity (SOR) is important to consider given its high prevalence in autism (over 50%; Baranek et al., 2006) and (separately) its association with FND symptoms (McCombs et al., 2024; Ranford et al., 2020).

Relatedly, reduced habituation (a slower or weaker reduction in response to repeated stimuli) has been demonstrated in studies with autistic people and people with FND (Merchie & Gomot, 2023; Voon et al., 2010). Although currently there are no publications assessing sensory processing or habituation in autistic people diagnosed with FND, in one study of autistic participants, tactile hypersensitivity was a risk factor for symptoms of weakness and paraesthesia (abnormal sensations such as tingling, prickling or ‘pins and needles’) (Nisticò et al., 2022). Sensory processing and habituation should be future research priorities in understanding the autism–FND association, with attention to consistent terminology and precise identification of the specific stage of processing under study.

The high co-occurrence of Hypermobility Spectrum Disorder (HSD) with autism (Nisticò et al., 2022) is another important biological consideration, given HSD occurs at a higher relative prevalence in FND and is an independent likelihood factor for FSs (Koreki et al., 2022). Putative mechanisms underlying an association between HSD and FS implicate a ‘neuroconnective endophenotype’ (Bulbena-Vilarrasa et al., 2025) where collagen and glycoprotein alterations are associated with dysregulated autonomic control, impaired interoception and functional neural connectivity atypicalities (Koreki et al., 2022). However, data are lacking as to the likelihood of autistic individuals with (vs. without) HSD or motor impairment developing FND.

##### Psychological and social factors

Cognitive models of FND highlight attentional disturbance and reduced mental flexibility as key mechanisms (Millman et al., 2023). Somatic hypervigilance (a heightened focus on bodily sensations) is central, with many FND triggers involving events that draw attention to the body, such as physical trauma, illness or medical procedures (Stone et al., 2009). This is particularly relevant to autism, where cognitive and attentional inflexibility are reported as more prevalent (Demetriou et al., 2018; Lage et al., 2024) and related to restricted and repetitive behaviours (Miller et al., 2015). Such patterns may heighten bodily focus or reduce the ability to disengage from distressing sensations, increasing vulnerability to functional symptoms in autistic people, given the key role of attention in FND pathophysiology.

Affect and emotion dysregulation, influential to internalised and bodily attention (Jeon et al., 2020), also feature in cognitive models of FND. In autism, elevated emotion dysregulation and maladaptive regulation strategies are common, shaped by factors such as alexithymia (difficulty identifying and describing one’s own emotions, and distinguishing emotional feelings from bodily sensations; Gaigg et al., 2018; Preece et al., 2017), intolerance of uncertainty, sensory reactivity and social adversity (Bruggink et al., 2016; Greaves, 2024; McDonald et al., 2024). These difficulties may limit coping and resilience, impairing allostasis and thereby compounding susceptibility to FND.

Where emotion is heightened, bodily processes or functions which should be automatic or not requiring conscious thought, such as motor planning and initiation, can be disrupted, reflected in aberrant amygdala-SMA connectivity (Pick et al., 2019; Sojka et al., 2018; Voon et al., 2010). This relates to the concept of ‘choking under pressure’, where athletes experience temporary impairment in high-pressure scenarios (Vine et al., 2016).

Similarly, psychological factors are linked to social factors affecting the autistic population. Higher prevalences of adverse life events and trauma (Pfeffer, 2016; Reuben et al., 2021; Roberts et al., 2015; Rumball, 2019; Stewart et al., 2022), social isolation, socioeconomic hardship and unemployment (Davies et al., 2023; Grace et al., 2022; Griffiths et al., 2019) are well-documented in the autistic population and are also risk factors for FND (Mavroudis et al., 2024). Autistic people may be more vulnerable to adverse experiences due to social communication differences, sensory sensitivities and societal misunderstanding, with twin studies suggesting this vulnerability partly reflects shared genetic factors linking neurodevelopmental traits to increased risk of maltreatment (Dinkler et al., 2017).

The true prevalence of trauma in FND is debated (ranging from 15% to 77%; Pun et al., 2020) and was previously overstated, although trauma is clearly a risk factor for FND (with odds ratios of 3–5; Ludwig et al., 2018). In parallel, there is a growing body of research on trauma and autism, reporting higher rates of traumatic stress and post-traumatic stress disorder (PTSD), with an estimated prevalence of 16%–44% compared to 4%–5% in the general population (Andrzejewski et al., 2024; Haruvi-Lamdan et al., 2020; Rumball et al., 2021). Furthermore, evidence is growing of events not meeting the DSM-5 PTSD definition of trauma (such as sensory overload, loss of routine and distressing healthcare encounters) triggering traumatic stress or PTSD in autistic individuals (Rumball et al., 2020).

Traumatic events influence FND-relevant constructs such as sensory reactivity, interoception and hypervigilance, and the severity of early-life physical abuse has been shown to correlate with alterations in insula, amygdala and motor connectivity in patients with FND (Diez et al., 2021; Lim & Young, 2025). It is also important to consider, especially concerning the autistic population, the social context such adverse experiences occur in, with reduced social support and barriers to healthcare, and how this complex situation may amplify distress and reduce resilience.

In summary, the interplay of neurobiological traits, cognitive differences and psychosocial adversity in autism aligns with a diathesis-stress framework for understanding FND (Weber et al., 2022), where cumulative stress triggers functional symptoms in an already vulnerable system.

#### The neuropsychological framework as applied to autism and FND

##### Interoception, emotion and alexithymia

Two connected constructs relevant to FND pathophysiology have gained considerable attention in the autism literature: interoception and alexithymia (Pick et al., 2019; Shah et al., 2016; Williams et al., 2022). Interoception refers to the brain’s moment-to-moment modelling of the internal physiological state of the body. It entails the sensory signalling, perceptual processing and psychological representation of sensations from internal bodily organs at conscious and unconscious levels (Khalsa et al., 2018; Murphy et al., 2017; Tsakiris & Critchley, 2016).

Interoception, like exteroception, has been modelled within the frameworks of active inference and allostasis (Barrett, 2017; Seth, 2013), meaning the brain models and infers the body’s internal state to anticipate and adapt to environmental demands by implementing changes (via the autonomic nervous system) that influence the interoceptive state. Emotions have been proposed as manifestations of interoceptive inference (Seth & Friston, 2016), that is, the phenomenological experience arising from the brain’s high-level (top-down) predictions about the causes of internal bodily states, integrated with exteroceptive information in the anterior insula (Seth, 2013).

Relatedly, Barrett’s (2017) Theory of Constructed Emotion frames emotions as conceptual constructions used to make such interoceptive predictions and support the brain’s allostatic goal of energy regulation. According to this view, emotion concepts, learned through experience, language and culture, enable the brain to categorise interoceptive inputs in context-specific ways, thereby shaping the emotional experience.

In these models of emotion, alexithymia is understood as a manifestation of dysfunctional interoceptive inference (Seth & Friston, 2016; Sowden et al., 2016) or a lack of emotion concepts to make such inferences (Jungilligens et al., 2022). This proposal is supported by findings from Trevisan et al.’s (2019) meta-analysis, which reported a moderate-to-strong negative association between alexithymia and interoception in studies with autistic participants.

##### Interoception and alexithymia in FND and autism

The pathophysiological role of interoception in FND is well supported (Drane et al., 2020; Pick et al., 2019; Sojka et al., 2021), implicating abnormal inferences about the state of and demands on the body, resulting in experiences of illness, fatigue and dissociation (Butler et al., 2021; Jungilligens et al., 2022; Sojka et al., 2024; Tsakiris, 2017). The inclusion of impaired emotion construction within this abnormal inferential process is highly relevant to FND because it places emotional and bodily information within one conception, thereby escaping mind–body dualism (Sojka et al., 2018).

Relatedly, alexithymia, which is prevalent in both FND (35%–75%) (Demartini et al., 2014; Gulpek et al., 2014) and autism (40%–60%) (Shah et al., 2016; Williams et al., 2022), has a proposed mechanistic role in FND. Goldstein and Mellers (2006) observed, in patients with FSs, autonomic activation without conscious experience of emotion or the ‘panic attack without panic’ phenomenon. Jungilligens et al. (2022) extended this as a failure to construct an emotion category, wherein interoceptive signals are not successfully integrated into a consciously accessible emotional category.

The proposed implications of FND as a failure to apply emotion concepts (impaired interoceptive inference), or a lack of emotion concepts (alexithymia), are that inaccurate or less granular emotion categories result in misattribution of arousal and physical sensations as illness or dysfunction (Jungilligens et al., 2022). Impaired allostasis and chronic energy mismanagement are proposed as key consequences of this, supported by fatigue being the most reported symptom by patients with FND (Butler et al., 2021).

Interoceptive differences in autism are supported by meta-analyses of functional neuroimaging studies of the insula cortex (Di Martino et al., 2009; Guo et al., 2024), a key interoceptive hub (Evrard, 2019) and by experimental data. Garfinkel et al. (2016) reported that their group of autistic participants displayed an ‘impaired ability to objectively detect bodily signals alongside an over-inflated subjective perception of bodily sensations’, with similar findings in autistic children later shown by Palser et al. (2018). Supporting this, Williams et al.’s (2022) meta-analysis reported that autistic participants showed significantly reduced heartbeat counting performance (interoceptive accuracy) but higher confidence in their interoceptive abilities (interoceptive sensibility).

This discrepancy between performance and confidence has been conceptualised as the interoception trait prediction error (ITPE), which has been shown to predict anxiety in autism (Garfinkel et al., 2016). In FND, higher ITPE scores strongly predict dissociation (Koreki et al., 2020) (defined as a disruption in the integration of consciousness, memory, identity, emotion, perception or bodily awareness; American Psychiatric Association, 2022) and have been found to correlate negatively with the integrity of white matter tracts originating from the bilateral insula and temporoparietal junction (TPJ), regions critical for integrating multisensory information and supporting body ownership (Maurer et al., 2016; Salgues et al., 2021; Sojka et al., 2021).

These findings implicate the ITPE as a mechanistic factor at the autism–FND interface, that is, difficulties accurately predicting the state of the body, and (in the context of the Theory of Constructed Emotion) findings of high rates of alexithymia would suggest the misattribution, or lack of, emotion concepts to infer the state of the body, allowing illness concepts to dominate affective feelings.

Interoception studies in autism are not uniform in their findings, however (highlighted by the Williams et al., 2022 meta-analysis), reflecting measurement confounders as well as population heterogeneity. Also, the evidence for impaired emotion concept granularity as a causal factor in FND remains speculative and requires empirical support.

In summary, the high prevalence of alexithymia in autism, combined with its link to atypical interoception and evidence of a greater likelihood of interoceptive differences in autistic individuals, suggests these factors could be vital in understanding the autism–FND relationship. It emphasises the need for studies investigating FND prevalence in autistic groups without alexithymia or raised ITPE.

Equally important is the evaluation of more objective markers of interoceptive processing in autism, such as the heartbeat-evoked potential (HEP) (a neural response linked to cardiac signals and considered a cortical marker of interoceptive processing), which has been reported as significantly reduced in FS (Elkommos et al., 2023; Koreki et al., 2020) and also in autism by Cari-Lène (2019), although these findings still require replication.

## The association between autism and FND in a computational framework

### Predictive processing and autism

A computational approach to understanding how attentional, sensory and motor processing differences in autism might increase the risk of FND might centre on atypical precision weighting, indicating altered assignment of confidence to sensory input relative to prior beliefs. This shift can modify how sensory evidence influences perception and action, potentially affecting not only lower-level sensory processing but also higher-order functions such as multisensory integration, emotional perception, agency and body ownership.

When reviewing predictive processing theories of autism, Chrysaitis and Seriès (2023) summarised these theories as positing an over-reliance on bottom-up rather than top-down information, described as the imbalance hypothesis (i.e., imbalance of influence between priors, sensory data and prediction errors). Their review found that the slight majority of studies did not find strong support for the imbalance hypothesis in autism; however, more than one-third of the studies did show some evidence of reduced influence of priors in autistic individuals.

To quote Brock (2012), ‘it is probably unwise to speak of “autistic perception” as if there were only one mechanism. The Bayesian account allows for the possibility that similar atypicalities of perception may arise for different reasons in different autistic individuals’.

### Predictive processing and autism and FND

Predictive processing theories are not constrained to a single tendency in precision weighting across the cortical processing hierarchy (Goodwin et al., 2025), that is, ascending levels from primary sensory input to higher-order cognitive representations. In this sense, disorders associated with overly weighted priors, such as FND, are not incompatible with research in autism suggesting higher-weighted sensory data or prediction errors.

In predictive coding accounts of FND, the core problem is thought to lie in the generation of high-level body-related priors. In the original formulation (Edwards et al., 2012), a triggering event (physical or psychological) is proposed to induce an abnormal symptom-related prior that is afforded excessive precision through heightened body-focused attention, thereby overwhelming lower levels of the predictive hierarchy. However, these models have yet to clarify why certain individuals are more susceptible to this process than others, beyond general predisposing or precipitating factors.

While motor research in autism suggests high precision weighting on proprioceptive afferents (Lidstone & Mostofsky, 2021), interoceptive research suggests difficulties filtering out (down-weighting or attenuating) sensory noise (unreliable data) at lower processing levels (Folz et al., 2024; Quattrocki & Friston, 2014; Van de Cruys et al., 2014), giving rise to highly weighted prediction errors, in line with subjective experiences of difficulty ignoring stimuli and contextualising raw sensory data, somatic hypervigilance or objective findings of raised ITPE.

In the HIPPEA (High, Inflexible Precision of Prediction Errors in Autism) theoretical framework (Van de Cruys et al., 2014, 2017), prediction errors are given too much precision (weight), regardless of context, meaning that random noise is treated as meaningful within the hierarchy, or there is difficulty disentangling signal from noise across contexts. This may prompt higher-level systems to generate maladaptive body-related priors as a compensatory response to reduce uncertainty (or prediction error or ‘free energy’; Friston et al., 2016).

**Figure 1.**
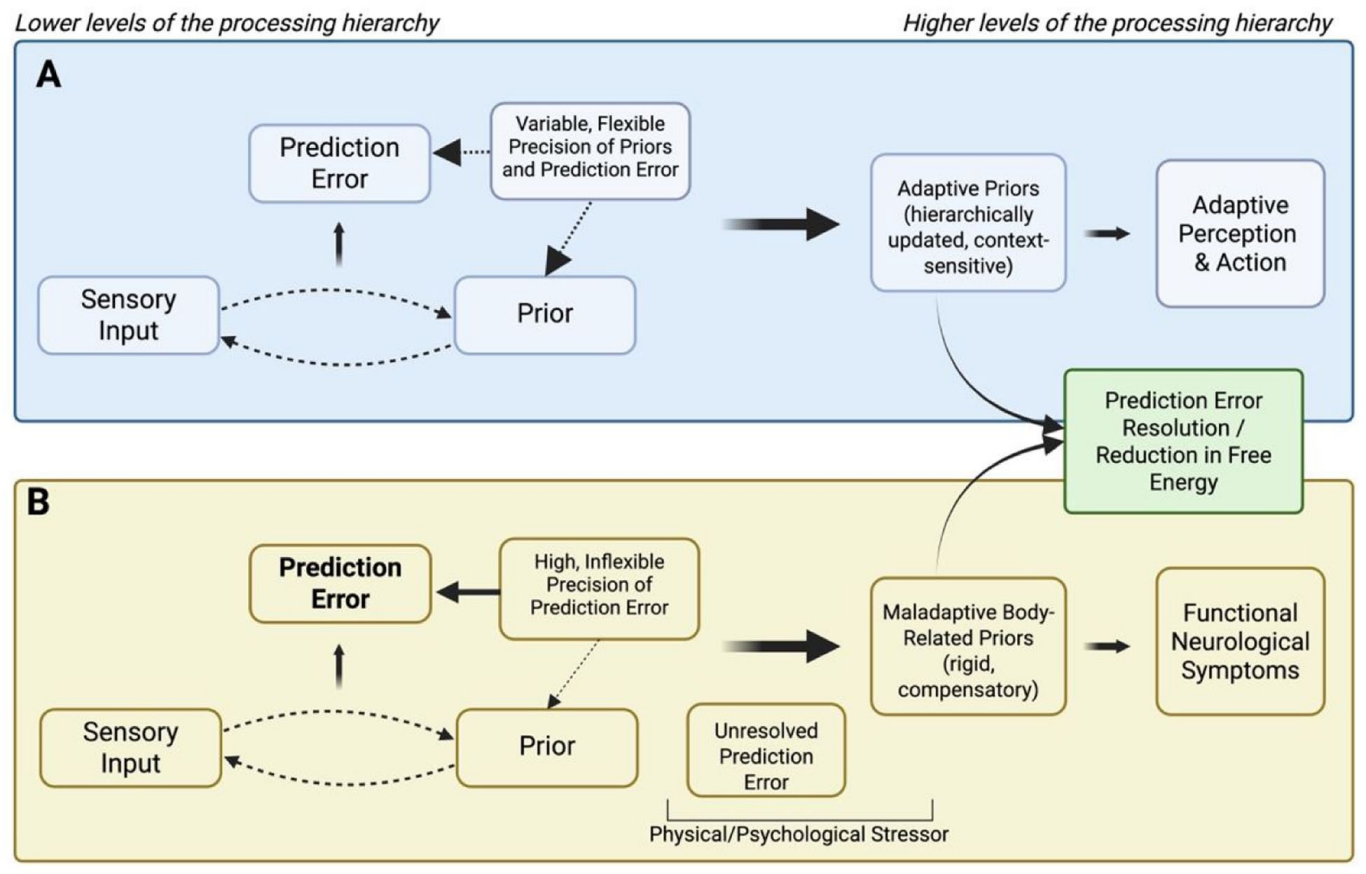
Predictive processing accounts for adaptive perception (A) and a theoretical model of vulnerability to functional neurological symptoms in autism, in line with the HIPPEA (High, Inflexible Precision of Prediction Errors in Autism) theory (Van de Cruys et al. 2014) (B). (A) In typical predictive processing, sensory input and priors interact dynamically, with prediction errors (discrepancies between expectation and input) used to update priors in a flexible, context-sensitive way. Precision (the weight given to priors or prediction errors) is variable and adaptively tuned, enabling prediction error minimisation, reduction of free energy, and coherent perception and action. (B) In the classical model of FND (not illustrated, see Edwards et al 2012.), a triggering event (physical or psychological) generates salient prediction errors that are afforded excessive precision through abnormal body-focused attention. This leads to the emergence of maladaptive, symptom-related priors that dominate lower-level sensory evidence and generate functional symptoms. Here, we propose that in autism predictive hierarchies may be especially vulnerable to this process as (in line with the HIPPEA framework, see Van de Cruys et al. 2014) lower levels of the hierarchy are already characterised by high, inflexible precision of prediction errors, increasing the likelihood of unresolved prediction errors and creating strong pressure for compensatory maladaptive priors. These priors then rigidly explain away persistent errors, but at the cost of generating functional neurological symptoms. *Source*. Created in BioRender by Cole, R. (2025) https://BioRender.com/px12o9i.

That is, when the system cannot attenuate or flexibly reweigh imprecise yet highly salient sensory signals, it may escalate the interpretation to higher-order cognitive levels to impose coherence on otherwise unreliable input (Harding et al., 2024) (see Figure 1). From this perspective, autistic individuals who find it difficult to down-weight noisy sensory input may be particularly prone to forming strongly weighted maladaptive priors that lead to functional symptoms, an account that parallels predictive coding models of abnormal beliefs developing to ‘explain away’ prediction errors caused by aberrant salience (Goodwin et al., 2025; Sterzer et al., 2018).

In summary, applying a predictive processing framework to the autism–FND intersection suggests that disrupted precision weighting manifests as an internal attentional misalignment, driven not only by external events but also by common differences in interoceptive processing in autism. The resulting maladaptive compensatory strategies may contribute to functional symptoms. However, this remains speculative, and further studies are needed to explore predictive processing paradigms in autistic individuals with a high functional symptom burden compared to those without.

## Important considerations

### Conceptualising the conceptualisation

Returning to the question of how to conceptualise the autism–FND association, several important considerations arise. Using the term ‘co-occurring’ or ‘co-morbid’ to understand FND and autism may even be a false premise to start with, as it makes two assumptions. First, it assumes distinct underlying mechanisms, which contrasts with inherent heterogeneity within autism and FND, and also the absence of unified psychophysiologies. Second, it assumes a categorical approach is correct, where autism and FND are conceptualised as distinct neuropsychological categories, with certain symptom combinations qualifying membership.

Simply put, do we have sufficient conceptual clarity? Is our initial exploratory approach susceptible to the limitations of the DSM’s exclusionary criteria, which do not reflect the dimensional or correlated nature of psychiatric phenomena? Furthermore, considering the high prevalence of anxiety in both autism and FND, should the question be framed as bivariate comorbidity (i.e., two diagnoses), trivariate (e.g., autism, FND and anxiety) or multivariate?

The Associated Liabilities Model (Krueger & Markon, 2006) offers a useful framework for conceptualising the autism–FND association. It proposes that each condition is linked to its own latent liability factor (e.g., Factor A for autism, Factor B for FND), and the degree of correlation between these factors determines the likelihood of co-occurrence. This allows for overlap in vulnerability, such as shared genetic or neurobiological traits related to sensory processing or alexithymia, without implying causation or a single underlying mechanism. It suggests that co-occurrence may arise from correlated liabilities, for example, ‘co-travelling’ traits of autism (e.g., alexithymia) rather than its core features, consistent with aetiological uncertainty.

Autism and FND may also co-occur due to different combinations of causes, some shared, some unique, that vary across individuals. Drivers of an association may vary between functional motor, sensory, cognitive or seizure disorders, as well as between autistic individuals with different degrees of trait clusters or in societies with varying levels of accommodation and ableism. This aligns with mechanistic pluralism and multifactorial causation, making a single explanatory mechanism for the overlap implausible. This carries similarities with an ‘increased vulnerability model’, which does not require a causality between autism and FND, just an elevation of risk (Krueger & Markon, 2006).

### A call to action for FND services

**Figure 2.**
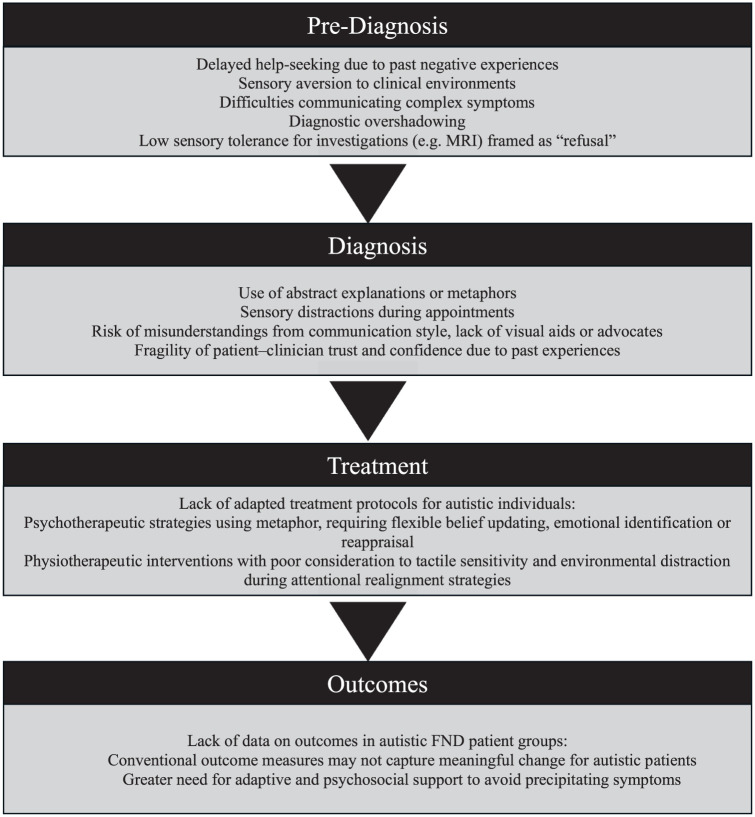
Potential challenges across the functional neurological disorder (FND) patient journey for autistic individuals.

Recognising the co-occurrence of autism and FND has important clinical implications. It highlights a potentially overlooked source of disability, stigma and barriers to healthcare in a population already facing healthcare inequalities (Raymaker et al., 2017). Understanding this association may help prevent misdiagnosis and missed diagnoses, enable earlier intervention and promote more tailored, effective treatment. It also prompts reflection on whether current FND pathways adequately serve autistic individuals, who may face distinct challenges and vulnerabilities throughout the clinical journey (see Figure 2), and whether outcomes valued by autistic people are being considered.

The growing evidence base of an autism–FND association and the known unmet healthcare needs in autistic patient groups (Raymaker et al., 2017) should serve as a call to action for FND multidisciplinary teams. Communication difficulties, sensory sensitivities, lack of appropriate accommodations and reduced healthcare self-efficacy can all hinder access to timely, coordinated care (Croen et al., 2015; Nicolaidis et al., 2013). Collectively, these barriers heighten the risk of misdiagnosis, unnecessary or intrusive investigations, dismissal of functional symptoms, and increased reliance on emergency services. FND services should therefore consider their practices to better meet the needs of autistic patients and reduce inequities in diagnosis, engagement and outcomes.

## Conclusion

Understanding a possible association between FND and autism requires embracing complexity, analysing nosology and adopting a transdiagnostic perspective. Neuropsychiatric disorders such as FND arise not from isolated factors but from a complex web of interactions that operate across multiple levels of analysis (Öngür & Paulus, 2025).

While predictive processing offers a compelling framework for understanding the potential links between FND and autism, it is essential not to become overly fixated on one level of understanding. Developmentally mediated and environmentally shaped differences in emotion processing, interoception, attention, multisensory integration and motor pathways are all important factors in considering the autism–FND intersection, while socioeconomic instability, barriers to healthcare in the context of morbidity and interpersonal adversity in the context of social isolation are of equal importance.

In societies that often overlook neurodivergent needs, autistic people commonly navigate external environments that are uncertain, unpredictable, distressing and potentially traumatising, with resulting sensory overwhelm, social adversity, systemic barriers and inequalities. This chronic extra requirement to predict, adapt and endure, in the context of lifelong differences in perceiving and acting in the world, translates as a significant allostatic burden and may result in forced maladaptive responses in the brain and body.

Autistic people with lived experience of FND should be meaningfully included at the centre of both academic inquiry and clinical service development. Their insights are essential to ensuring that research questions, diagnostic processes and therapeutic approaches reflect real-world needs, priorities and outcomes. Without their involvement, efforts to understand and address the autism–FND interface risk reproducing the very exclusions and misunderstandings they seek to remedy.

## Supplemental Material

sj-docx-1-aut-10.1177_13623613251393504 – Supplemental material for Exploring the autism and functional neurological disorder association: Considerations from biopsychosocial, neuropsychological and computational models Supplemental material, sj-docx-1-aut-10.1177_13623613251393504 for Exploring the autism and functional neurological disorder association: Considerations from biopsychosocial, neuropsychological and computational models by Richard H Cole, Lily Smythe, Mark J Edwards, Francesca Happé and Timothy R Nicholson in Autism
