# Supplementary material for: Exploring the autism and functional neurological disorder association: Considerations from biopsychosocial, neuropsychological and computational models
Source: Autism. 2025 Dec 17;30(2):269–84. doi: 10.1177/13623613251393504 (PMC12804432; doi:10.1177/13623613251393504)
Supplement: sj-docx-1-aut-10.1177_13623613251393504 – Supplemental material for Exploring the autism and functional neurological disorder association: Considerations from biopsychosocial, neuropsychological and computational models [file sj-docx-1-aut-10.1177_13623613251393504.docx]

**Glossary of Terms**

**Active inference:** A framework in which both perception and action serve to minimise prediction error. Rather than separating perception (updating beliefs) and action (controlling behaviour), active inference unifies them: perception updates internal beliefs to better predict sensory input, while action fulfils those predictions by changing the world or the body to bring sensory input in line with expected outcomes.

**Alexithymia:** Difficulty identifying and describing one’s own emotions, and distinguishing emotional feelings from bodily sensations. It does not mean a person lacks emotions, but rather struggles to put them into words. It is split into three dimensions:

1. Difficulty identifying feelings (DIF): Problems recognising and differentiating between emotional states and physiological arousal.
2. Difficulty describing feelings (DDF): Limited ability to articulate emotions to others.
3. Externally oriented thinking (EOT): A cognitive style characterised by a focus on external, concrete events rather than internal emotional experiences or imagination.

**Allostasis:** The anticipatory regulation of bodily states in which the brain predicts and adjusts physiological activity to meet expected demands.

**Autonomic regulation:** The processes by which the autonomic nervous system (ANS) automatically controls and adjusts internal bodily functions to maintain physiological stability (homeostasis). This includes the regulation of heart rate, blood pressure, respiration, digestion, temperature, and other visceral activities through coordinated activity of the sympathetic (activating) and parasympathetic (restorative) branches.

**Embodiment:** The integration of cognitive, emotional, and perceptual experience which contribute to the sense of being and having a body, and to the body being the medium through which one experiences and acts in the world. It encompasses the felt senses of body ownership and agency.

**Feigning:** The intentional production or exaggeration of physical or psychological symptoms; **different** from malingering (faking for external gain).

**Free Energy:** A measure of the mismatch between the brain’s predictions and sensory inputs, quantifying how well its generative model explains incoming data. By minimising free energy through updating predictions (perceptual inference) or acting to sample expected inputs (active inference), the brain reduces prediction error and maintains adaptive, self-organising regulation of its internal and environmental states.

**Functional Neurological Disorder:** A disorder characterised by genuine, involuntary neurological symptoms such as weakness, tremor, sensory loss, or non-epileptic seizures that are incongruent with recognised neurological disease and not explained by structural damage.

**Functional Somatic Syndromes:** Syndromes, such as fibromyalgia, irritable bowel syndrome, and chronic fatigue syndrome, characterised by persistent bodily symptoms without identifiable structural pathology. Though named by the body system affected, they share underlying neural processes involving pain perception, autonomic regulation, and visceral function.

**Heartbeat Evoked Potential:** An electroencephalographic (EEG) measure of cortical responses time-locked to the R-peaks of the electrocardiogram (ECG), reflecting the brain’s processing of afferent signals from the cardiovascular system. It is considered a neural marker of interoceptive processing, with HEP amplitude increasing during heightened attention to internal bodily states.

**Hypermobility Spectrum Disorder:** A group of conditions characterised by symptomatic joint hypermobility (JHM) (an increased range of motion of one or more joints beyond normal limits). While JHM is common and often benign, its association with pain, instability, or soft-tissue injury may indicate an underlying connective tissue abnormality. HSD lies on a continuum between generalised joint hypermobility and hypermobile Ehlers-Danlos syndrome (hEDS), encompassing individuals with symptomatic JHM who do not meet criteria for hEDS or another heritable or neuromuscular disorder.

**Interoception:** The brain’s moment-to-moment modelling of the body’s internal physiological state, involving the sensory signalling, perceptual processing, and representation of signals from internal organs at conscious and unconscious levels. Interoception enables the brain to anticipate and regulate bodily needs by adjusting physiological states via the autonomic nervous system (allostasis). Peripheral interoceptive signals ascend through the spinothalamic tract and cranial nerves to the thalamus and insular cortices, where they are integrated to support awareness of bodily state and self.

**Interoceptive accuracy:** An interoceptive dimension reflecting the objective ability to detect internal bodily signals, typically measured through tasks such as heartbeat counting or detection.

**Interoceptive sensibility:** the subjective belief or self-reported awareness of one’s internal bodily sensations, often assessed through questionnaires or confidence ratings on interoceptive tasks.

Interoceptive Trait Prediction Error: the mismatch between interoceptive accuracy and interoceptive sensibility (a dissociation between objectively measured and subjectively perceived interoceptive ability). It reflects impaired metacognitive insight into one’s interoceptive performance, meaning the individual’s self-evaluation of bodily awareness does not align with actual interoceptive accuracy.

**Mind-body dualism:** The philosophical view that the mind and body are fundamentally distinct entities; the mind as non-physical (thought or consciousness) and the body as physical matter, interacting but separable in nature.

**Precision weighting:** The process by which the brain adjusts the relative influence (gain) of prior expectations versus sensory prediction errors according to their estimated reliability. It modulates perceptual inference by tuning the neural gain of prediction error signals, and it is mechanistically linked to neuromodulators such as dopamine and acetylcholine. Through adaptive precision weighting, the brain filters out unreliable or noisy information, optimising the balance between expectation and evidence to support efficient perception, learning, and action.

**Predictive perception:** The theoretical framework wherein the brain constructs hierarchical generative models, shaped by prior experience, to predict internal (bodily) and external (environmental) states. Incoming sensory signals (exteroceptive, proprioceptive, interoceptive) are compared against these predictions (priors), and mismatches generate prediction errors that update the model and shape perceptual experience.

**Prior:** A pre-existing belief or expectation within the brain’s generative model about the likely causes of sensory input, formed through past experience. Priors guide perception and inference by shaping how incoming sensory signals are interpreted and how prediction errors are resolved.

**Sensorimotor processing:** The integration of sensory input with motor output, enabling the brain to interpret incoming sensory information and generate coordinated movements. It underlies perception, action, and bodily awareness by continuously linking what is sensed with how the body responds.

**Somatic hypervigilance:** A heightened focus on bodily sensations, involving excessive attention to normal physiological signals or minor changes in the body. This increased monitoring can amplify symptom perception and contribute to the experience of physical discomfort or distress in the absence of structural disease.

**Theory of Constructed Emotion:** The theory proposing that emotions are not innate, fixed biological reactions but are constructed by the brain through the integration of interoceptive, sensory, and conceptual information. According to this framework, the brain continually predicts and interprets bodily states within context, using prior experiences and conceptual knowledge to categorise affective sensations as specific emotions (e.g., “anger” or “fear”).
